# Pontine tubercular abscess: A rare presentation of CNS tuberculosis masquerading as glioma in a child

**DOI:** 10.1002/ccr3.9179

**Published:** 2024-07-14

**Authors:** Bikash Chandra Adhikari, Sangam Rouniyar, Ujjawal Roy, Bishal Kunwor, Ashmina Lama

**Affiliations:** ^1^ Nepalese Army Institute of Health Sciences Kathmandu Nepal; ^2^ Kathmandu Medical College and Teaching Hospital Kathmandu Nepal

**Keywords:** brain abscess, craniotomy, glioma, tuberculosis

## Abstract

Tuberculous brain abscess (TBA) in a child was initially misdiagnosed as glioma. Two craniotomies, abscess drainage, and anti‐tubercular therapy led to recovery. Pontine TBA, though rare and atypical, can have better outcome with timely intervention.

## 1 INTRODUCTION

Brain abscesses are rare infections localized in the brain parenchyma, with an incidence rate ranging from 0.3 to 1.3 cases per 100,000 individuals per year.^1^ Most intracranial abscesses are localized to the cerebrum, particularly in the frontal and temporal lobes. Causes for brain abscesses are a direct spread of infections from the paranasal sinuses and middle ear caused by oropharyngeal organisms and indirectly due to the hematogenous spread of organisms.^2^ Abscesses in the brainstem are even rare, accounting for less than 1% of brain abscess cases.^1^

Tuberculous brain abscess (TBA) is a rare presentation of central nervous system (CNS) tuberculosis. An encapsulated collection of pus drained from brain parenchyma with a viable number of tubercular bacilli indicates TBA.^3^ The surgical therapy of TBA involves excision of the abscess and stereotactic aspiration of pus to craniotomy.^4^

## 2 CASE HISTORY (PRESENTATION AND EXAMINATION)

A 14‐year‐old female child presented with a 3‐day history of right‐sided hemiparesis. A neurological assessment revealed complete awareness and orientation. The functioning of the cranial nerves, visual field, and visual acuity were all normal. Weakness of the right upper and lower limbs was present (Medical Research Council grade 2 out of 5). Deep tendon reflex testing showed 1+ grading for the biceps and supinator tendon.

## 3 METHODS (DIAGNOSTIC ASSESSMENT AND TREATMENT)

A complete blood count (CBC) showed a white blood cell (WBC) count of 15 × 10^3^/μL, hemoglobin (Hb) count of 11.3 g/dL, hematocrit of 34.0%, neutrophil 86.0%, and lymphocytes 10.5%. Platelet and red blood cell (RBC) counts were normal. The patient's blood culture was negative for *Staphylococcus aureus*, *Streptococcus pyogenes* as well as gram‐negative bacteria. Serology for human immunodeficiency virus (HIV), hepatitis B virus (HBV), and hepatitis c virus (HCV) was negative (Table 1).

**TABLE 1:** Blood investigation findings at the time of admission and a month of treatment.

Blood report (unit)	During admission	After a month of treatment	Reference range
White blood cells (WBC) (10^3^/μL)	15	5.5	4–11
Neutrophils (%)	86	53.3	40–80
Lymphocytes (%)	10.5	37.3	20–40
Basophils (%)	0.0	0.0	0–2
Eosinophils (%)	0.7	5.8	1–6
Monocyte (%)	2.7	3.6	2–10
Platelets count (10^3^)	357	353	150–450
Hematocrit (%)	34.0	36.1	36–47
Hemoglobin (Hb) (g/dL)	11.3	12.6	12–15
RBC count (10^6^/μL)	4.26	4.58	3.6–5
Mean cell volume (MCV) (fL)	80	79	80–100
RDW‐CV (%)	12.4	15	11–15
RDW‐SD (fL)	36	42	36–47
Mean platelets volume (fL)	7.4	7.0	7.5–11.5
Mean cell hemoglobin concentration (g/L)	33.4	35.0	31–35
Mean cell hemoglobin (pg)	26.6	27.6	26–34

**FIGURE 1:**
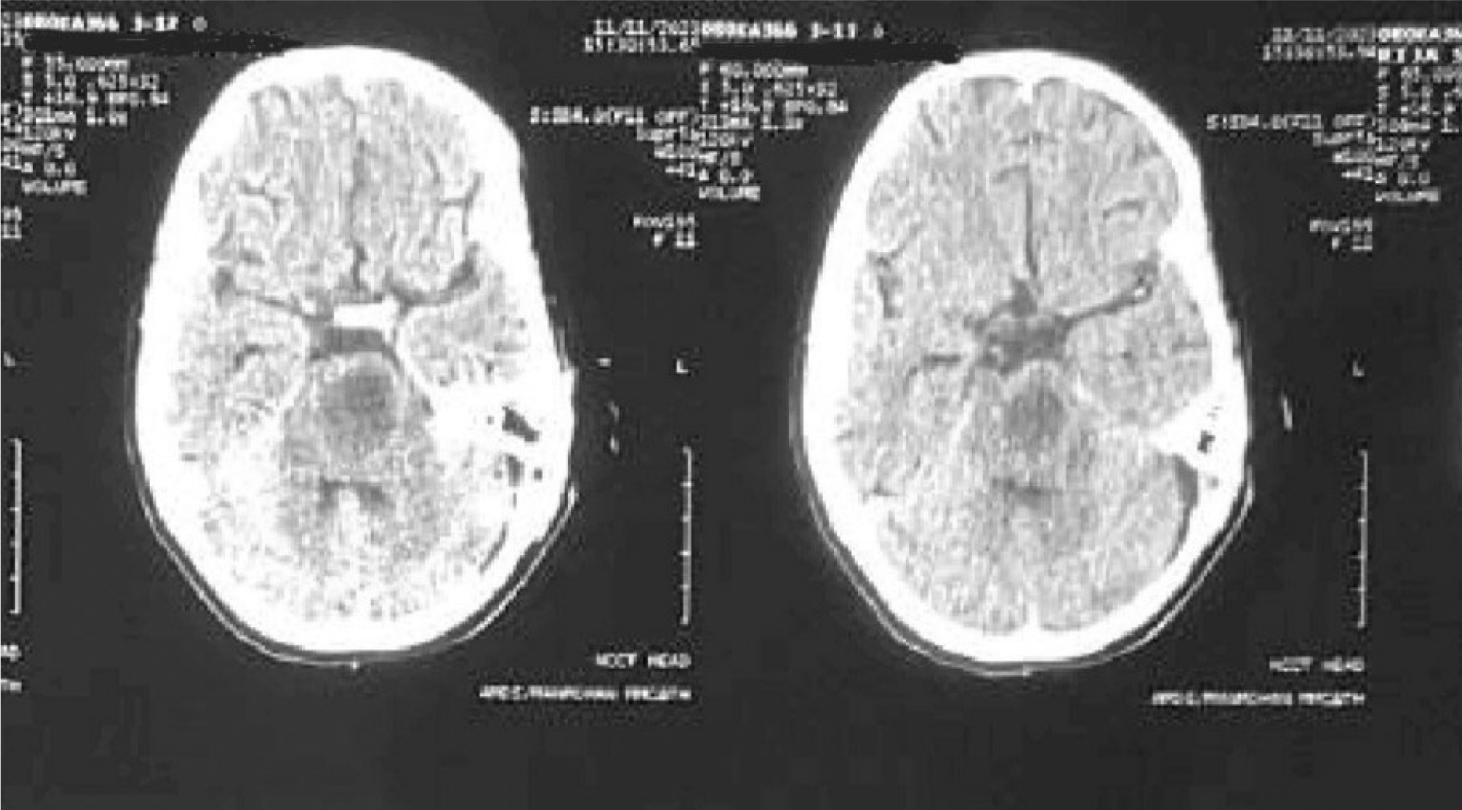
CT scan showing an ill‐defined hypo‐dense area in the left area of the brain stem predominantly pons.

**FIGURE 2:**
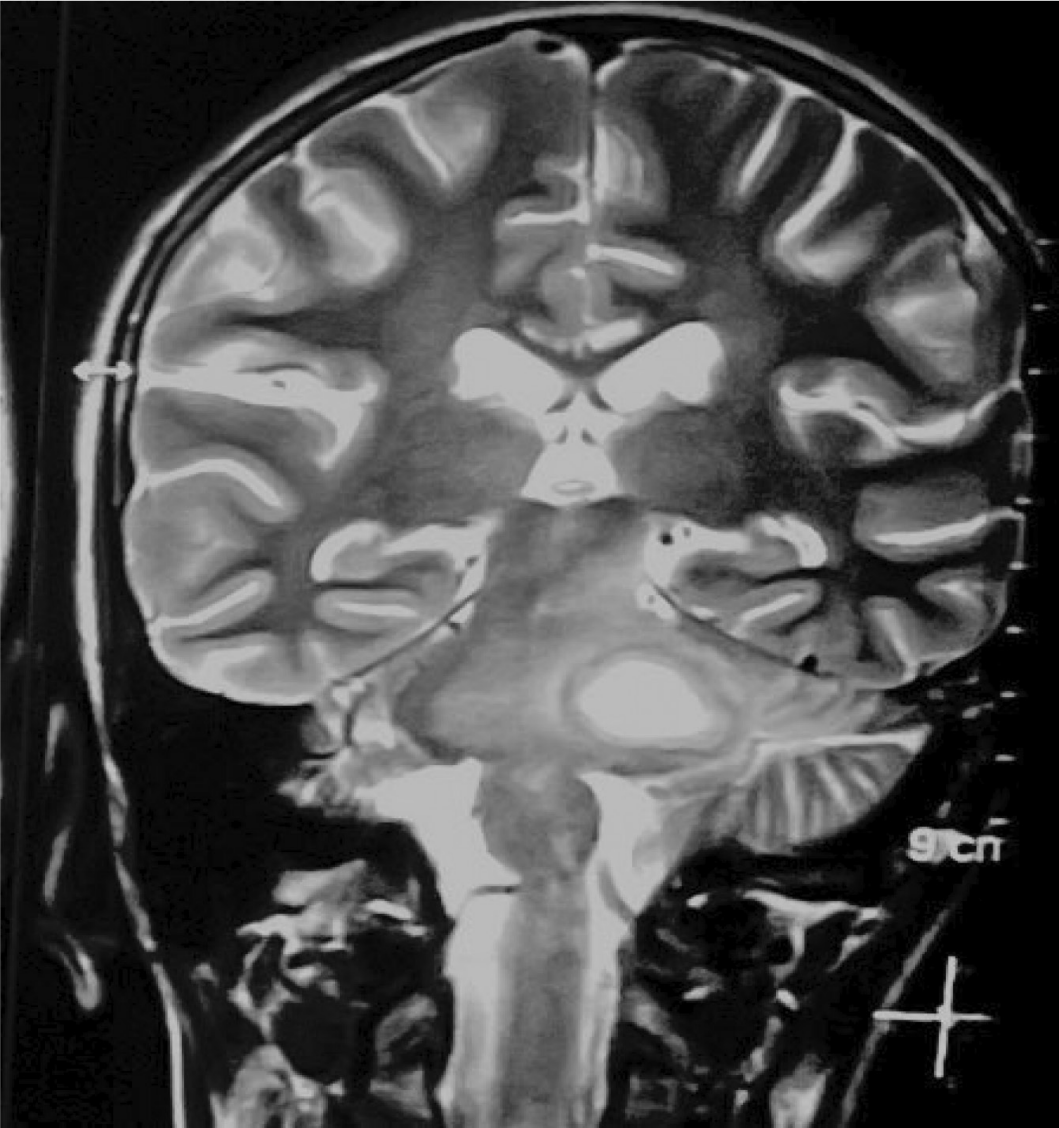
Coronal view of contrast‐enhanced T2 weighted MRI of head showing rim‐enhancing cystic lesion in left pons and within the left side of cerebellopontine angle.

**FIGURE 3:**
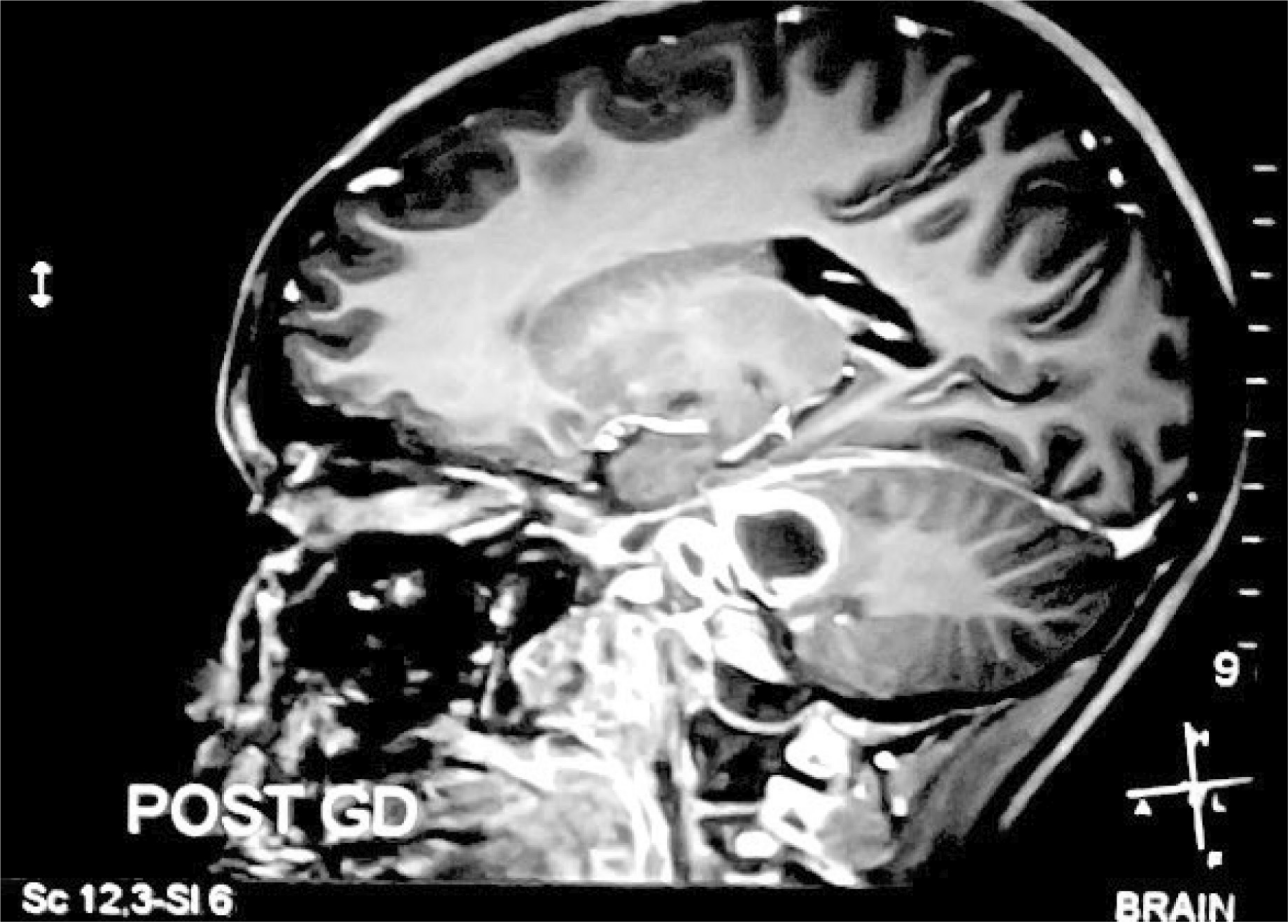
Sagittal view of contrast‐enhanced T1 weighted MRI of head showing rim‐enhancing cystic lesion in left pons and within the left side of the cerebellopontine angle.

The initial plain computed tomography (CT) scan of the head was performed which showed ill‐defined hypodensity in the left posterior limb of the internal capsule, midline pons, and left pontocerebellar fibers of cerebellum suggesting infarction and encephalo‐myelitic changes (Figure 1). Further investigation with magnetic resonance imaging (MRI) revealed a cystic lesion affecting the left cerebellar peduncle and left area of the pons (Figures 2 and 3). The fifth, sixth, seventh, and eighth cranial nerves were found to be bilaterally normal by the Constructive Intervention in Steady State (CISS) protocol technique. These results led to the initial suspicion of glioma.

The left retro sigmoid craniotomy was done to drain the abscess from pontine area.

Following a left retro sigmoid craniotomy, an abscess was found on the left side of the midbrain (predominantly pons) and the cerebellopontine angle. The pontine abscess was verified based on these findings. After the abscess was drained, pus along with blood was sent for additional investigations.

The patient was started on clindamycin, ceftriaxone (as empirical therapy), codeine, and dexamethasone. Ziehl–Neelsen staining of pus showed acid‐fast bacillus (AFB) and polymerase chain reaction (PCR) for *Mycobacterium tuberculosis* was positive.

Anti‐tubercular therapy (ATT) was started on the same day, the pontine tubercular abscess was diagnosed as mentioned in the diagnosis section. The patient was regularly followed up for 40 days.

**FIGURE 4:**
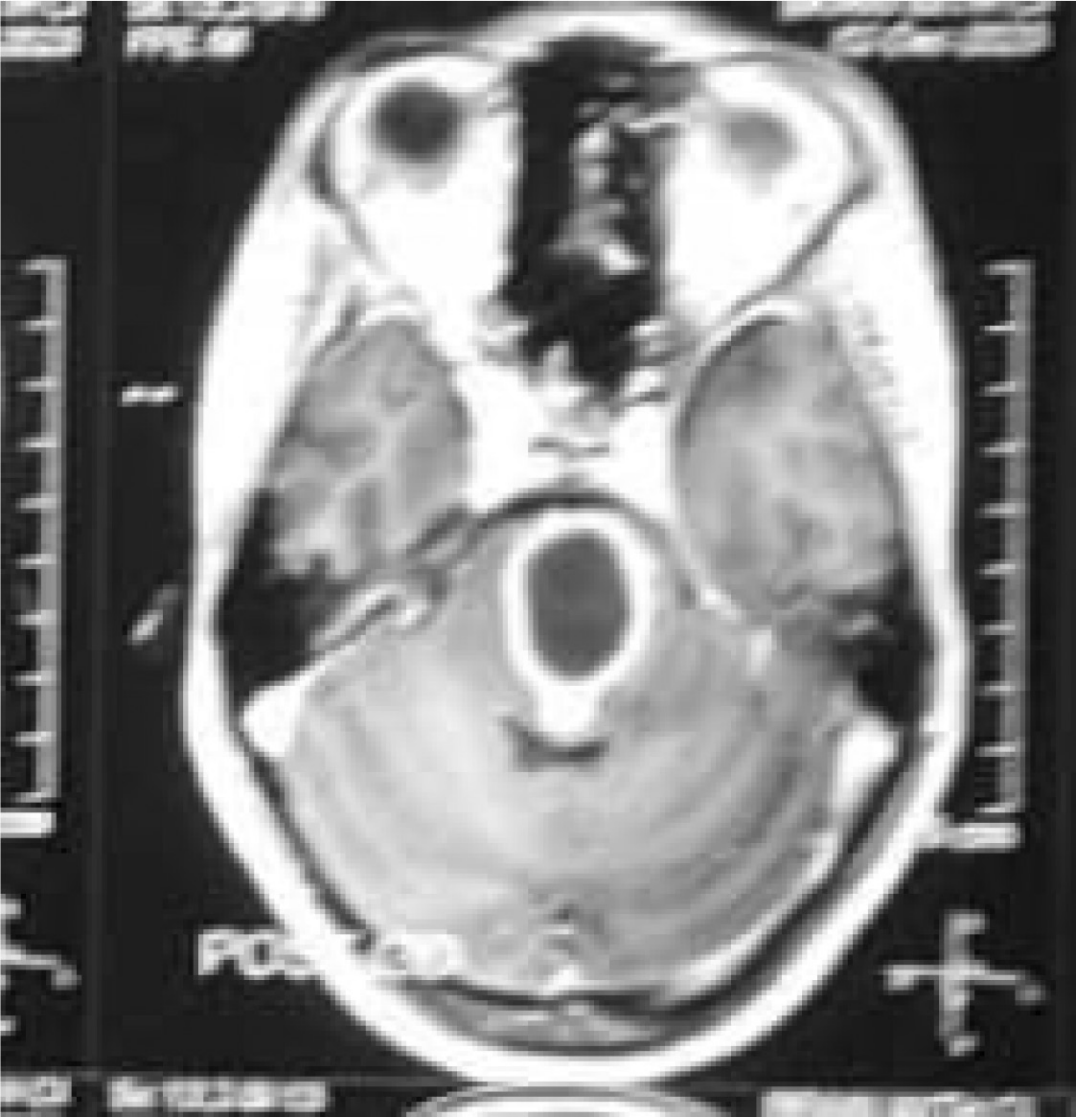
Contrast‐enhanced MRI in transverse section showing cystic lesion in the pontine area.

A repeat MRI was performed which showed a well‐defined, encapsulated, and thick‐walled area measuring 2.6 × 2.4 × 2.0 cm (volume = 6.5 cc) in size localized to the pons (Figure 4). There was another similar area measuring 11.9 × 8.2 mm in size within the left side of the cerebellopontine angle. A repeat CBC report showed normal WBC count, Hb level, hematocrit, neutrophils, and lymphocytes. Left retro sigmoid craniotomy was re‐performed for abscess drainage. ATT was continued and her condition had significantly improved. Following her improved health, she was sent to physiotherapy for right‐sided hemiparesis. As she recovered her motor power, she was discharged from the hospital and began routine follow‐up. She has now finished her full ATT course and is doing well on her follow‐ups.

## 4 DISCUSSION

Brain abscesses are rare infections of the brain parenchyma, with an incidence rate ranging from 0.3 to 1.3 cases per 100,000 individuals per year.^1^ A retrospective investigation carried out by Nathoo et al., found that only three out of 973 individuals with brain abscesses had abscesses in the brainstem.^5^ This signifies that tubercular pontine abscess is even rarer.

The presence of pus within the brain and demonstration of AFB on staining, culture, or PCR are criteria for TBA.^6^ Our patient fulfilled the criteria of TBA with the presence of AFB on staining and PCR‐positive result in the pus sample collected during craniotomy for diagnosis of tubercular abscess.

TBA patients typically exhibit localized neurological symptoms at first. Examining the cerebrospinal fluid may reveal pleocytosis with elevated protein and a PCR test may reveal the presence of mycobacteria in a significant number of cases.^7^ In our case, the patient had an acute onset of right‐sided hemiparesis with no history of typical symptoms.

A hypodense lesion encircled by an augmenting ring is typically visible on CT scans.^3^, ^7^ There may be connected surrounding edema. In our case, CT showed ill‐defined hypodensity in the left posterior limb of the internal capsule, midline pons, and left pontocerebellar fibers of the cerebellum. MRI shows a hyperintense central area with a hypointense rim on T2 weighted images and a hypo‐intense on T1 weighted with peripheral rim enhancement.^6^, ^8^ MRI findings were similar in our case with additional perilesional edema. This surrounding brain edema and low gliosis initially led us to suspicion of glioma. TBA was confirmed only after the left retro sigmoid craniotomy and AFB staining of pus.

TBA caused by *Mycobacterium tuberculosis* is treated with multidrug ATT. This therapy includes bactericidal first‐line agents such as isoniazid (H), rifampicin (R), pyrazinamide (Z), and bacteriostatic drug ethambutol (E). Streptomycin (S) is included in a case of drug resistance. Duration of treatment is 2 months with H, R, Z, E and an additional 8–10 months with H and R.^9^ Pyridoxine was also prescribed to prevent peripheral neuropathy. Corticosteroids are used for elevated intracranial pressure or severe cerebral edema.^10^ Dexamethasone was used in our case. Differential diagnoses that may need to be considered are solitary cysticercus granuloma, cystic glioma, and chronic pyogenic abscess.

Simple puncture, continuous drainage, fractional drainage, repeated aspiration through burr holes, and total excision of the abscess are the surgical options that might prove to be beneficial in the case of TBA. Total excision might be helpful in cases of multilocular non‐communicating and thick‐walled abscesses.^11^ We performed retro sigmoid craniotomy for abscess drainage in our patient. With abscess drainage, ATT, and supportive physiotherapy our patient showed great clinical improvement.

## 5 CONCLUSION

In conclusion, TBA is a rare manifestation of brain abscess. Our case posed a diagnostic challenge as the initial investigation with CT scan suggested us glioma. However, through surgical intervention and microbiological investigations, TBA was finally diagnosed. Abscess drainage and multi drug ATT were greatly helpful in improving the patients' condition. This case highlights the importance of considering TBA in differential diagnosis of brain abscesses, particularly in the region where TB is endemic.

## AUTHOR CONTRIBUTIONS

**Bikash Chandra Adhikari:** Conceptualization; resources; supervision; validation; visualization; writing – original draft; writing – review and editing. **Sangam Rouniyar:** Conceptualization; supervision; writing – original draft; writing – review and editing. **Ujjawal Roy:** Conceptualization; writing – original draft; writing – review and editing. **Bishal Kunwor:** Conceptualization; resources; supervision; validation; visualization; writing – original draft; writing – review and editing. **Ashmina Lama:** Conceptualization; writing – original draft; writing – review and editing.

## FUNDING INFORMATION

This article did not receive any grants.

## CONFLICT OF INTEREST STATEMENT

The authors declare no conflicts of interest.

## CONSENT

Written informed consent form was obtained from the patient's father, the patient being a minor, to publish this report in accordance with the journal's consent policy.

## PATIENT PERSPECTIVE

The patient and her family members were anxious about the patient's condition. They were properly counseled and assured that she would get better.

## Data Availability

All the findings are present within the manuscript.
